# A cross sectional study of non-medical use of prescription opioids and suicidal behaviors among adolescents

**DOI:** 10.1186/s40621-021-00329-7

**Published:** 2021-07-19

**Authors:** Nate Wright, Marizen R. Ramirez

**Affiliations:** Division of Environmental Health Sciences, School of Public Health, 1215-1 Mayo, 420 Delaware Street SE, Minneapolis, MN 55455 USA

**Keywords:** Adolescent, Analgesics, opioid, Suicidal ideation, Suicide, attempted, Pain

## Abstract

**Background:**

Non-medical use of prescription opioids (NMUPO), defined as taking an opioid in a manner or dose other than prescribed, taking someone else’s, or for the feelings that it produces, has been reported by 5 to 20% of adolescents, and is associated with a two fold increase in suicidal behaviors among adolescents. Adolescents with long-term health problems (LTHP) have poorer mental health and may seek NMUPO for pain relief that is not obtained through standard care. For this study, we measured the association between NMUPO and suicidal behaviors, and further assessed effect modification by LTHP hypothesizing the association between NMUPO and suicidal behaviors was stronger for adolescents with LTHP.

**Findings:**

For students with LTHP, 13.5% reported suicide ideation, while 8.0% of students without LTHP reported suicide ideation. For suicide attempt, 4.4% of students with LTHP reported a suicide attempt, while 2.1% of students without LTHP reported a suicide attempt. The proportion of students who reported suicide ideation and attempts increased with higher occasions of NMUPO. Adjusted logistic regression models found increased odds of suicide ideation (OR (95% CI): 1–5 occasions: 2.3 (2.0–2.6); > 6 occasions: 2.7 (2.2–3.8)) and suicide attempts (OR (95% CI): 1–5 occasions: 3.2 (2.7–3.7); > 6 occasions: 4.1 (3.1–5.3)) for students who reported NMUPO. An interaction term for NMUPO and LTHP was then added to the models. Effect modification was not present on the multiplicative scale. On the additive scale, evidence of effect modification was observed: higher risk was indicated for students with LTHP versus no LTHP for both suicide ideation (Predicted risk (95%CI): > 6 occasions NMUPO, LTHP: 0.24 (0.18–0.29); No LTHP: 0.16 (0.13–0.18)) and attempt (Predicted risk (95%CI): 1–5 occasions NMUPO, LTHP: 0.08 (0.07–0.10); No LTHP: 0.05 (0.05–0.06); > 6 occasions NMUPO, LTHP: 0.11 (0.07–0.15); No LTHP: 0.06 (0.05–0.08)).

**Conclusions:**

The results affirmed that NMUPO is associated with suicidal behaviors among adolescents. A pattern also emerged of higher risk of suicidal behaviors for those with LTHP who reported NMUPO. Areas of further inquiry should explore chronic medical or pain conditions as possible modifying pathways that could exacerbate the effects of NMUPO on suicidal behaviors specific to an adolescent population.

## Introduction

Non-medical use of prescription opioids (NMUPO) is defined as taking an opioid in a manner or dose other than prescribed, taking someone else’s prescription opioid, even if for a legitimate medical reason, or taking prescription opioids for the feelings that it produces (National Institute on Drug Abuse 2019). Common reasons for NMUPO include the relief of physical pain, the feelings they produce, to relax or relieve tension, and to help with feelings or emotions (Center for Behavioral Health Statistics and Quality, R. T. I. International 2015). Prescription opioids are also a means for suicide, the second leading cause of death for adolescents and young adults (Centers for Disease Control and Prevention 2018). Recent research has found that NMUPO is significantly associated with a 1.5 to two times increased risk of suicidal behaviors in adolescents (Ashrafioun et al. 2017; Baiden et al. 2019; Zullig et al. 2015; Amanda and Keith 2014).

NMUPO is theorized to be associated with suicidal behaviors for a number of reasons – opioids may disinhibit risky or impulsive suicidal behavior (Ilgen et al. 2016); NMUPO is associated with the onset or recurrence of depression, a known risk factor for suicide (Scherrer et al. 2016); NMUPO may be a proxy for untreated pain that is a risk factor for suicidal behavior (Scherrer et al. 2016; Ford and Perna 2015; Kuramoto et al. 2012); and adolescents with comorbid substance use disorder are significantly more likely to attempt suicide (Amanda and Keith 2014). Based on three studies in the US, a rather wide range of adolescents reported NMUPO; approximately 5 to 20% reported lifetime misuse (Zullig et al. 2015; Havens et al. 2011; McCabe et al. 2019a). Examining NMUPO in adolescents continues to be a priority, especially as a risk factor for suicidal behaviors and potential means for suicide. In Minnesota, suicide is the leading cause of death for youth 12–18 years of age (Centers for Disease Control and Prevention W 2018).

Furthermore, understanding gateways to NMUPO will focus prevention efforts. Of relevant concern is a potentially high-risk group of adolescents with long-term health problems (LTHP). Because of the undertreated or uncontrolled pain or symptoms associated with a diagnosed condition, adolescents with LTHP may seek NMUPO for pain or symptom relief that is not obtained through standard care (Ilgen 2018; Koenig et al. 2015). Pain and chronic conditions often start in adolescence, with an increasing prevalence of these conditions that continues into adulthood (Battaglia et al. 2020). Similar trends are observed in NMUPO, which also peaks in young adulthood (McCabe et al. 2019b). Those with LTHP have poorer mental health, including higher risk for depression, decreased sense of belonging and coping strategies (Dean-Boucher et al. 2019), increased feelings of hopelessness, isolation, burdensomeness, and lack of control over life circumstances (Dean-Boucher et al. 2019). Therefore, the relationship between NMUPO and suicidal behaviors may be modified by the presence of LTHP.

In this study, NMUPO and its association with suicidal behaviors will be examined in adolescents. Additionally, the modifying effect of LTHP on this association will be analyzed. This study used Minnesota Student Survey measures that inquire about past year use of NMUPO and past year suicidal behaviors among Minnesota youth in 2016. It was hypothesized that NMUPO will be positively associated with both suicide ideation and attempt, and this association will be stronger for adolescents with LTHP compared to those without LTHP. Within the context of the current opioid overdose epidemic and the steady rise in suicide mortality, this knowledge is crucial to understand the complex association between these behaviors.

## Methods

### Survey and participants

This study utilized anonymous, school-based, cross-sectional data from the 2016 Minnesota Student Survey (MSS). A census methodology targeting all schools and students in Minnesota was used for obtaining responses from fifth, eighth, ninth, and eleventh grade students. The survey resulted in 85% of school districts participating, representing students from public, non-public, charter, and tribal schools. By grade, approximately 66% of fifth graders, 73% of eighth graders, 71% of ninth graders, and 61% of eleventh graders participated.

The 2016 MSS data contained responses from 168,733 students. Fifth grade students were removed from analysis because suicide-related questions were not asked, which resulted in 126,868 student responses. Additionally, responses with a history of suicide attempt or suicide ideation of more than 1 year were excluded from analysis to limit temporality of NMUPO and suicidal behaviors to within the same time frame for the purpose of more closely aligning to the hypotheses for NMUPO as an acute exposure for suicidal behaviors. The final study sample was composed of 8th, 9th, and 11th grade students who provided responses to questions about past year suicide ideation and suicide attempts, and occasions of past year NMUPO (*N* = 112,275).

### Measures

#### Outcome

The outcomes of interest were past year suicide attempt (“Have you ever actually attempted suicide?”) and suicide ideation (“Have you ever seriously considered attempting suicide?”). An affirmative response to either question was counted as past year suicide attempt or ideation. The outcomes were not mutually exclusive and participants could report one or both outcomes.

#### Exposure

The exposure was self-reported NMUPO (“During the last 12 months, on how many occasions (if any) have you used pain relievers, such as Oxycodone, OxyContin (oxy), Percocet, Percodan, Vicodin, or others, that were not prescribed for you or that you ONLY took to get high?”). The survey-defined response categories ranged from zero to 20 or more occasions in the past year. Categorization of NMUPO was evaluated through descriptive and regression analyses. Categories were collapsed where odds ratios were similar between groups with decisions supported through the use of Akaike and Bayesian Information Criterion (AIC/BIC) to assess model fit. As a result, NMUPO was recoded and analyzed as a three-level exposure with none (0 occasions), medium (1–5 occasions), and high (6 or more occasions) to characterize the distribution of responses.

#### Effect modifier

The association was further explored with LTHP (“Do you have any physical disabilities or long-term health problems? Long-term means lasting six months or more.”) as an effect modifier. The association between NMUPO and suicidal behaviors was compared between those with and without LTHP.

#### Covariates

A directed acyclic graph (DAG) was developed and used to identify covariates that were included in the final models to control for potential confounding. These were sex (i.e., male or female), school grade (i.e., eight, ninth, or eleventh grade), receipt of free or reduced-price lunch (i.e., proxy indicator for socioeconomic status), self-reported alcohol use in the past 30 days (i.e., 0 days, 1–2 days, 3 or more days), and self-reported depressive symptoms (i.e., feeling down, depressed, or hopeless) in the last 2 weeks (i.e., yes or no).

### Analysis

Crude and adjusted logistic regression models were performed. Adjusted models examining the association between NMUPO and suicidal behaviors controlled for LTHP, sex, grade, free or reduced-price lunch, depressive symptoms in the past 2 weeks, and alcohol use in the past 30 days. An interaction term for NMUPO and LTHP (i.e., NMUPO*LTHP) was then added to the regression models, while still controlling for potential confounders, to examine the modifying role of LTHP. Effect modification by LTHP was evaluated on both the multiplicative and additive scales. Less than 9% of observations were missing data for LTHP, NMUPO, and suicidal behavior; these records were excluded from regression analyses. Clustering within schools was also examined and found to be small with no meaningful impact on regression results.

## Results

The study sample contained an approximately even number of 8th and 9th grade respondents, with a slightly lower number of 11th grade respondents. The majority were white (80.3%), and the average age was 14.8 years. In all, 27.2% of students reported receiving free or reduced price lunch, a proxy measure for socioeconomic status. Depressive symptoms were reported by 33% of students, while about 11% of students reported any alcohol use in the past 30 days. Finally, 14.1% of students reported LTHP (Table 1).

**Table 1:** Student demographics asked about suicide attempts and suicide ideation, Minnesota Student Survey

Grade	*N* (%)
8	40,982 (36.5)
9	40,261 (35.9)
11	31,032 (27.6)
Race^a^	*N* (%)
American Indian/Alaskan Native	6170 (5.5)
Asian	8951 (8.0)
Black, African, or African American	11,330 (10.0)
Native Hawaiian/Other Pacific Islander	1243 (1.1)
White	90,169 (80.3)
Age	Mean (SD)
	14.8 (1.3)
Gender	*N* (%)
Male	58,833 (52.6)
Female	53,132 (47.5)
Free/reduced Price Lunch	*N* (%)
Yes	30,607 (27.2)
Depression	*N (%)*
Yes	37,021 (33.0)
Physical/long-term Health Problem	*N* (%)
Yes	15,803 (14.1)
Alcohol Use (in the past 30 days)	*N* (%)
0 days	91,431 (81.4)
1–2 days	7698 (6.9)
3 or more days	4674 (4.2)

^a^Race categories are not mutually exclusive

Among students, 8.8% reported suicide ideation in the past year. For students with LTHP, 13.5% reported suicide ideation, while 8.0% of students without LTHP reported suicide ideation. The proportion of students who reported suicide ideation increased with higher NMUPO. At every level of NMUPO, students with LTHP had a higher proportion of suicide ideation than students without LTHP (Table 2).

**Table 2:** Suicide ideation and occasions of NMUPO^a^ in the past year by long-term health problems^b^

	Suicide ideation *N* = 112,275		Suicide Ideation - No LTHP *N* = 93,033		Suicide Ideation – LTHP *N* = 15,803	
NMUPO	Yes (*N*)	%	Yes (*N*)	%	Yes (*N*)	%
0 occasions	8124	8.2	6328	7.5	1766	12.6
1–5 occasions	568	29.0	426	27.1	136	36.6
> 6 occasions	180	33.2	125	29.8	55	45.8
Total	8872	8.8	6879	8.0	1957	13.5
*p*-value		< 0.0001		< 0.0001		< 0.0001

^a^Non-medical use of prescription opioids was defined as use of pain relievers that were not prescribed for you or that you only took to get high

^b^Long-term health problem was defined as any physical disabilities or long-term health problems lasting 6 months or more

A total of 2.4% of students reported a suicide attempt in the past year. For students with LTHP, 4.4% reported a suicide attempt, while 2.1% of students without LTHP reported a suicide attempt. The proportion of students who reported suicide attempts was higher for students who reported NMUPO. Students with LTHP who reported NMUPO had a higher proportion of suicide attempts than students without LTHP (Table 3).

**Table 3:** Suicide attempt and occasions of NMUPO^a^ in the past year by long-term health problems^b^

	Suicide Attempt *N* = 112,275		Suicide Attempt - No LTHP *N* = 93,033		Suicide Attempt – LTHP *N* = 15,803	
NMUPO	Yes (*N*)	%	Yes (*N*)	%	Yes (*N*)	%
0 occasions	2062	2.1	1524	1.8	533	3.8
1–5 occasions	280	14.3	196	12.4	82	22.3
> 6 occasions	96	17.7	64	15.3	32	26.5
Total	2438	2.4	1784	2.1	647	4.4
*p*-value		< 0.0001		< 0.0001		< 0.0001

^a^Non-medical use of prescription opioids was defined as use of pain relievers that were not prescribed for you or that you only took to get high

^b^Long-term health problem was defined as any physical disabilities or long-term health problems lasting 6 months or more

Adjusted logistic regression models found that the odds of suicide ideation for students who reported 1–5 occasions of NMUPO were 2.3 (95% CI: 2.0–2.6) times higher and for students who reported 6 or more occasions of NMUPO were 2.7 (95% CI: 2.2–3.8) times higher than students who reported 0 occasions of NMUPO. The odds of suicide attempt for students who reported 1–5 occasions of NMUPO were 3.2 (95% CI: 2.7–3.7) times higher and for students who reported 6 or more occasions of NMUPO were 4.1 (95% CI: 3.1–5.3) times higher than students who reported 0 occasions of NMUPO (Table 4).

**Table 4:** Adjusted associations between suicide ideation/suicide attempt and NMUPO

	Suicide Ideation			Suicide Attempt		
	OR	95% CI	*p*-value	OR	95% CI	*p*-value
0 occasions	Ref.	Ref.		Ref.	Ref.	
1–5 occasions	2.3	2.0–2.6	< 0.0001	3.2	2.7–3.7	< 0.0001
> 6 occasions	2.7	2.2–3.8	< 0.0001	4.1	3.1–5.3	< 0.0001

Note: Model controlled for LTHP, sex, grade, SES, alcohol use, and depression

An interaction term for NMUPO and LTHP (i.e., NMUPO*LTHP) was then added to the regression models, while still controlling for potential confounders. The interaction terms were not statistically significant, indicating no effect modification on the multiplicative scale for suicide ideation or suicide attempt. Still, a pattern emerged where students with LTHP who reported 6 or more occasions of NMUPO had higher odds of suicide ideation (No LTHP: OR = 2.5 (1.9–3.2); LTHP: OR = 3.6 (2.3–5.5)) and suicide attempt (No LTHP: OR = 3.8 (2.8–5.2); LTHP: OR = 4.7 (2.9–7.5)) compared to those without LTHP (Tables 5 and 6).

**Table 5:** Effect modification for LTHP on the multiplicative scale for the association between NMUPO and suicide ideation

NMUPO	No LTHP		LTHP		
	OR	95% CI	OR	95% CI	*p*-value
0 occasions	Ref.	Ref.	1.4	1.3–1.5	< 0.0001
1–5 occasions	2.3	2.0–2.6	2.2	1.7–2.8	0.61
> 6 occasions	2.5	1.9–3.2	3.6	2.3–5.5	0.16

**Table 6:** Effect modification for LTHP on the multiplicative scale for the association between NMUPO and suicide attempt

NMUPO	No LTHP		LTHP		
	OR	95% CI	OR	95% CI	*p*-value
0 occasions	Ref.	Ref.	1.7	1.5–1.9	< 0.0001
1–5 occasions	3.2	2.6–3.8	3.2	2.4–4.3	0.87
> 6 occasions	3.8	2.8–5.2	4.7	2.9–7.5	0.49

Note: Models controlled for sex, grade, SES, alcohol use, and depression

Evidence of effect modification by LTHP was found on the additive scale. For suicide ideation, no differences were found for students who reported 1–5 occasions of NMUPO, but students with LTHP who reported 6 or more occasions of NMUPO had higher risk of suicide ideation (No LTHP: 0.16 (0.13–0.18); LTHP: 0.24 (0.18–0.29)). For suicide attempt, higher risk was found for students with LTHP who reported 1–5 occasions (No LTHP: 0.05 (0.05–0.06); LTHP: 0.08 (0.07–0.10)) and 6 or more occasions (No LTHP: 0.06 (0.05–0.08); LTHP: 0.11 (0.07–0.15)) of NMUPO compared to students without LTHP (Tables 7 and 8).

**Table 7:** Effect modification for LTHP on the additive scale for the association between NMUPO and suicide ideation

NMUPO	No LTHP		LTHP		
	Predicted Risk	95% CI	Predicted Risk	95% CI	*p*-value
0 occasions	0.08	0.079–0.082	0.11	0.102–0.110	< 0.0001
1–5 occasions	0.15	0.136–0.162	0.18	0.152–0.205	0.80
> 6 occasions	0.16	0.131–0.180	0.24	0.182–0.289	0.07

**Table 8:** Effect modification for LTHP on the additive scale for the association between NMUPO and suicide attempt

NMUPO	No LTHP		LTHP		
	Predicted Risk	95% CI	Predicted Risk	95% CI	*p*-value
0 occasions	0.02	0.019–0.020	0.03	0.028–0.033	< 0.0001
1–5 occasions	0.05	0.046–0.062	0.08	0.066–0.102	0.08
> 6 occasions	0.06	0.048–0.080	0.11	0.072–0.148	0.09

Note: Models controlled for sex, grade, SES, alcohol use, and depression

## Discussion

The present study addresses a complex public health phenomenon of both increasing suicide and opioid-involved overdose deaths. Along with previous research (Ashrafioun et al. 2017; Amanda and Keith 2014; Ilgen et al. 2016; Scherrer et al. 2016; Ford and Perna 2015; Kuramoto et al. 2012; Rockett et al. 2014), these results provide evidence that suicidal behaviors and NMUPO are related. In this study of Minnesota youth, the odds of suicide ideation were two to three times higher and suicide attempt were three to four times higher among youth who reported NMUPO than youth who did not report NMUPO. These results are consistent with prior research examining this topic, and the magnitude of the association is also similar. Adolescents who report NMUPO have been found to have an estimated 1.5 to two times increased risk of suicidal behaviors relative to adolescents who do not report NMUPO (Ashrafioun et al. 2017; Baiden et al. 2019; Amanda and Keith 2014; Ford and Perna 2015).

The association between NMUPO and suicidal behaviors was also hypothesized to be modified by LTHP. No evidence of effect modification by LTHP was found on the multiplicative scale. However, an additive interaction was observed; the highest risks of suicidal behaviors were found among those who had LTHP and highest use (i.e., 6 or more occasions) of NMUPO. Effect modification on the additive scale is most relevant for public health because it provides information on how best to target prevention efforts since the results indicate how many more people would experience an outcome due to the exposure in one subgroup versus another. These results suggest that report of both LTHP and NMUPO substantively increase risk for suicidal behaviors. NMUPO may provide those with LTHP a way to cope with the stress of lacking support amid chronic medical conditions, and for pain or symptom relief that is not obtained through standard care. Suicidal behaviors are also associated with LTHP and chronic conditions, with individuals diagnosed during adolescence at increased risk for subsequent development of suicidal behaviors (Dean-Boucher et al. 2019). Taken together, these results call for increased screening for substance use and mental health concerns, particularly for those with LTHP, along with proper care management and support to adolescents who are diagnosed with LTHP.

Initiation of NMUPO may also be as a result of treatment for acute health conditions, such as an injury. For example, prescription opioid use may begin with a prescription following an injury, surgery, or dental procedure (Harbaugh et al. 2018; Schroeder et al. 2019). Since our results found increased risk for suicide ideation and attempts for those without LTHP who reported NMUPO, future investigations that examine the origins of NMUPO should also focus on acute health conditions that involve opioids.

There are limitations with the present study. The cross-sectional data represent prevalence at a point in time, with the inability to determine causation. This is also a school-based survey requiring that students attend school the day of the survey to participate. Absent students, who may be at higher risk for poor health and risky behaviors, are not included in the results, potentially limiting generalizability and biasing the results. However, over 60% of students did participate in the statewide survey. Despite the survey anonymity, the questions examined ask about sensitive or illegal behavior, which may result in social desirability bias. The NMUPO question is unique because it asks for the number of occasions of use in the past year, instead of the number of days. Occasions, however, is a vague term that may be understood differently by students, perhaps limiting the generalizability of the responses. Finally, LTHP, as defined by the survey, includes a variety of physical and medical conditions, such as chronic conditions (e.g., diabetes or asthma), mental health diagnoses (e.g., depression or ADHD), as well as physical disabilities. This heterogeneous group likely consists of conditions that may or may not involve prescription drug use, and as a result could diminish the magnitude of associations.

The present study found a significant association between NMUPO and suicide ideation and attempt, along with evidence for LTHP as an effect modifier. Emphasis is needed on the reduction and judicious prescribing of opioids, as well as the promotion of non-opioid pain management alternatives. Further research is also needed to explore specific chronic medical or acute pain conditions to reveal unique modifying pathways that could exacerbate the effects of NMUPO on suicidal behaviors. The broad category of LTHP utilized in this study did not allow for this level of detail to be examined. Nevertheless, adolescents with LTHP or newly diagnosed chronic conditions must be supported as treatment begins and symptoms are managed, which includes screenings for mental health and substance use. Additionally, future research should attempt to elucidate reasons for NMUPO and the source of prescription opioids for adolescents.

## Data Availability

The data that support the findings of this study are available from the Minnesota Department of Health but restrictions apply to the availability of these data, which were used under license for the current study, and so are not publicly available. Data are however available from the Minnesota Department of Health upon reasonable request. Additional information and user agreement and data request form can be found here: https://www.health.state.mn.us/data/mchs/surveys/mss/index.html.

## Abbreviations

MSS: Minnesota Student Survey
NMUPO: Non-medical use of prescription opioids
LTHP: Long-term health problems
DAG: Directed acyclic graph
